# Fermented Foods and Beverages of Ethiopia: Substrates, Traditional Preparation Techniques, and Nutritional Quality

**DOI:** 10.1002/fsn3.71887

**Published:** 2026-05-13

**Authors:** Limenew Abate Worku, Rakesh Kumar Bachheti, Vikas Rana, Gyanesh Joshi, Archana Bachheti, D. P. Pandey

**Affiliations:** ^1^ Department of Chemistry, College of Natural and Computational Science Debre Tabor University Debre Tabor Ethiopia; ^2^ Centre of Molecular Medicine and Diagnostics, Saveetha Dental College & Hospitals, Saveetha Institute of Medical and Technical Sciences Saveetha University Chennai India; ^3^ Cellulose & Paper Discipline, Forest Products Division Forest Research Institute Dehradun Uttarakhand India; ^4^ Department of Environment Science Graphic Era (Deemed To Be University) Dehradun Uttarakhand India; ^5^ Department of Chemistry Govt Degree College Dehradun Shahar Suddhowala, Dehradun India

**Keywords:** fermented foods, nutritional quality, substrates, traditional preparation techniques

## Abstract

Fermented foods play a vital role in improving nutrition, flavor, and shelf life through microbial activity. In Ethiopia, several traditional fermented products such as injera, a sourdough flatbread prepared from teff flour; kocho, a bread‐like product derived from the enset plant; and tella, a traditional fermented beverage, represent essential components of the daily diet and cultural heritage. These foods are produced using diverse substrates and indigenous preparation techniques that have been transmitted across generations. This review explores the substrates, traditional preparation methods, and nutritional quality of Ethiopian fermented foods and beverages. Fermentation enhances nutrient bioavailability, improves digestibility, and contributes beneficial microorganisms that support gut health. Despite their nutritional and cultural importance, traditional fermentation practices face several challenges, including limited standardization, insufficient scientific documentation, regulatory gaps, and economic constraints affecting small‐scale producers. Variability in preparation methods may also lead to differences in product quality and consumer acceptance. Future sustainability will depend on improved research on microbial diversity and nutritional composition, the development of standardized and hygienic production practices, and the integration of these foods into modern dietary frameworks. Strengthening support for small‐scale producers, encouraging innovation, and increasing public awareness can further enhance food security, economic development, and the preservation of Ethiopia's rich fermentation heritage.

## Graphical abstract

Traditional Ethiopian fermented foods and beverages such as Injera, Kocho, and Tella provide important health benefits, including improved nutrient bioavailability, gut health, and digestibility. However, challenges such as lack of standardization, insufficient documentation, and economic constraints limit their wider development and commercialization.

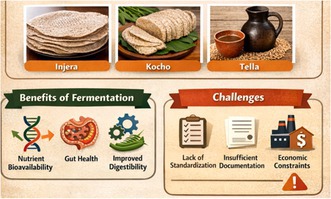

## 1 Introduction

Fermented foods result from metabolic processes that convert sugars and carbohydrates into organic acids or alcohol by microorganisms such as bacteria, yeast, and molds (Singh and Kumar 2025). This age‐old technique enhances flavor, texture, and nutritional value while also serving as a natural method of food preservation. The health benefits of fermented foods are well documented, including the presence of probiotics that improve gastrointestinal health, increased nutrient bioavailability, and contribution to overall wellness (Mukherjee et al. 2024).

Fermentation is deeply integrated into the traditional food system and is closely linked to cultural practices and day‐to‐day activities in Ethiopia. The country boasts a diverse array of fermented foods, reflecting its different agricultural resources and cultural heritages. For instance, *injera*, made from teff flour; *tella*, a traditional beer produced from barley, sorghum, or maize; and *ergo*, a fermented food product, are prominent examples (Mengesha et al. 2022). These delicacies are mainstays of Ethiopian cuisine, important at social events, and frequently served at religious and cultural festivals. Fermented foods serve similar purposes worldwide; regional variations exist in ingredients, production processes, and end products. Ethiopia's rich cultural heritage is emphasized by its variety of fermented dishes (Wedajo Lemi 2020). The majority of products are made utilizing naturally occurring bacteria and back‐slopping or spontaneous fermentation from plant‐based raw materials, mostly cereals and tubers, usually on a household scale (Horlacher et al. 2023). Fermentation offers a cost‐effective means of improving food safety and shelf life (Obafemi et al. 2022). It also reduces toxins, allergens, pathogenic microbes, and antinutritional factors, while enhancing mineral bioavailability and phytochemical content (Ram et al. 2020). It also increases the flavor, body, and aroma of the product, as well as introducing probiotic microorganisms that aid in the production of bioactive compounds beneficial for health (Sharifi‐Rad et al. 2020). Encouraging the production and consumption of fermented foods throughout Africa can help improve food security, offer diversified nutrient sources, and contribute to social development in impoverished communities (Materia et al. 2021).

Two of the most significant Ethiopian fermented foods are injera and kocho. Kocho is a bread‐like food item made from the corm and pseudostem of enset ( *Ensete ventricosum* ), a multipurpose crop that supports about 20 million Ethiopians, particularly in the South and Southwest regions of Ethiopia (Yemata and Fetene 2020). Baked kocho is a rich source of carbohydrates, nutrients, and lactic acid bacteria content and is often consumed with butter, spices, meat, lentils, or vegetables (Bosha et al. 2016; Seboka et al. 2023). Another staple is Ethiopian fermented flatbread, injera, made from teff ( *Eragrostis tef* ), a gluten‐free grain availabl**e** in both white and brown varieties (Neela and Fanta 2020). It is of great interest due to its excellent amino acid profile, high amount of minerals, phenolics, and complex carbohydrates (Neela and Fanta 2020; Yegrem et al. 2021). Both of these have great social and cultural importance in the Ethiopian food tradition. This review focuses on the history, preparation methods, nutritional value, and potential future applications of Ethiopian fermented foods in the context of food security, culture, and health.

## 2 Material and Method

This review follows the Preferred Reporting Items for Systematic Reviews and Meta‐Analyses (PRISMA) guidelines to ensure a structured and reproducible approach. Published research papers, review articles, proceedings, brief communications, and book chapters describing fermented food in Ethiopia served as the primary sources for the information in this article. A comprehensive literature search was performed across multiple electronic databases including Scopus, Web of Science, PubMed, Google Scholar, ScienceDirect, and African Journals Online (AJOL) from January 1980 to August 2025. Additional gray literature was sourced from Ethiopian institutional repositories, conference proceedings, and theses/dissertations from universities in Ethiopia. Search terms included “fermented food*” or “fermented beverage*” or “traditional fermentation” or “injera” or “kocho” or “tella” or “tej” or “ergo” or “ayib” or “borde” or “Shamita” or “korefe” or “cheka” or “keribo” or “areki” or “dhanaan” or “arrera” or “aguat” and (Ethiopia* OR “Ethiopian” OR Amhara OR Oromia OR Tigray OR Sidama OR Gurage) AND (microbial* OR bacteria or yeast or “lactic acid” or probiotic* or nutrition* or “nutritional value” or substrate* or “preparation method*” or “traditional processing”).

## 3 Fermented Milk‐Derived Products

Unpasteurized milk is consumed in many traditions, as traditional wisdom has taught people how to prepare and consume raw dairy products, including cream, butter, yoghurt, kefir, and cheese, for thousands of years (Visioli and Strata 2014). For dairy products, especially fermented products, consumers are drawn to their recognized nutritional and health benefits. The first time scientists became interested in the health effects of FM was in 1910 (Ailioaie and Litscher 2021; Mozaffarian 2016). Their effects are due to the bioactive constituents in milk and the bioactivities produced by probiotics as a result of the fermentation of dairy products (Santosa et al. 2006). Probiotics can be used directly as therapeutic or preventive agents against serious illnesses such as infectious diseases, atopic disorders, and tumors, and they can also alter several milk constituents. Through direct interactions with ingested microorganisms, probiotic effects, or microbial metabolites produced during fermentation, fermented functional foods provide health benefits (Doron and Gorbach 2006).

In Ethiopia, fermented dairy products are typically produced by allowing fresh milk to ferment naturally for two or more days in traditional vessels that have been pre‐smoked (Gonfa et al. 2001; Maleke et al. 2021). Indigenous people have long used smoking these traditional vessels with certain plant species (Sadik 2014). This process has both safety and sensory benefits: the smoke's antibacterial properties disinfect the containers, reduce spoilage microorganisms, extend shelf life, and enhance the aroma and taste of dairy products. Among Ethiopia's most important fermented dairy products are *Ergo* and *Ayib*.

### 3.1 Ergo (Spontaneously Fermented Whole Milk)

**FIGURE 1:**
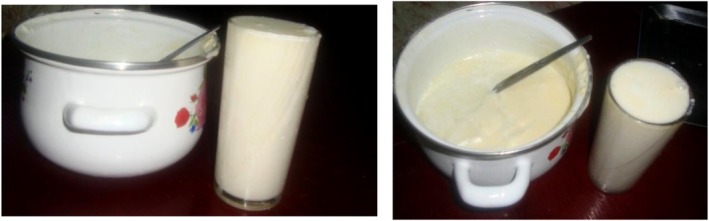
Ergo: A traditional fermented milk in Ethiopia.

Ergo (Figure 1) is the most widely consumed fermented milk product in Ethiopia. It is produced by allowing milk to ferment naturally at room temperature for two to 3 days, without the use of starter cultures (Karssa et al. 2024). The process is influenced by ambient temperature and incubation time, which vary by region (Assefa et al. 2008). *Ergo* resembles set yoghurt: thick, semisolid, white in color, smooth in texture, and mildly sour in taste. It may be consumed plain or with spices, though its flavor and consistency differ among ethnic groups depending on the spices and smoking materials used (Berhe et al. 2017).

During fermentation, lactic acid bacteria (LAB) dominate the microbial community, followed by yeasts and molds. Common LAB genera include *Lactobacillus*, *Lactococcus*, *Streptococcus*, *Leuconostoc*, and *Enterococcus*. These microorganisms enhance the bioavailability of nutrients, act as natural preservatives, and produce lactic acid, which contributes to product safety and health benefits (Gemechu 2015). Studies have confirmed the antimicrobial potential of LAB in Ergo, with isolates exhibiting antibacterial activity against human pathogens comparable to ampicillin (Bizuye et al. 2013; Tesfaye et al. 2011). This activity is attributed to the production of lactic acid and hydrogen peroxide, which significantly reduces enteropathogen counts during fermentation and storage. This highlights Ergo as a potential source of antimicrobial LAB with applications in food preservation and public health, especially against pathogens such as *Shigella*, *Listeria monocytogenes* , and *Staphylococcus aureus* (Fenta and Assefa 2017).

Traditional processing practices also enhance Ergo quality. Equipment is commonly washed and smoked with plant materials such as *Eucalyptus globulus* , *Cymbopogon martini* , and *Olea africana* to extend shelf life and improve flavor (Olaniran et al. 2024; Thakur et al. 2025). Smoking suppresses harmful microorganisms and spoilage, ensuring product safety. The reduced pH of Ergo after a full day of fermentation further inhibits pathogens. Typically, Ergo coagulates within 24 h and becomes increasingly sour with time, but it is most enjoyable at this stage due to its distinctive taste (Mulaw et al. 2025).

### 3.2 Ayib (Traditional Ethiopian Cottage Cheese)

**FIGURE 2:**
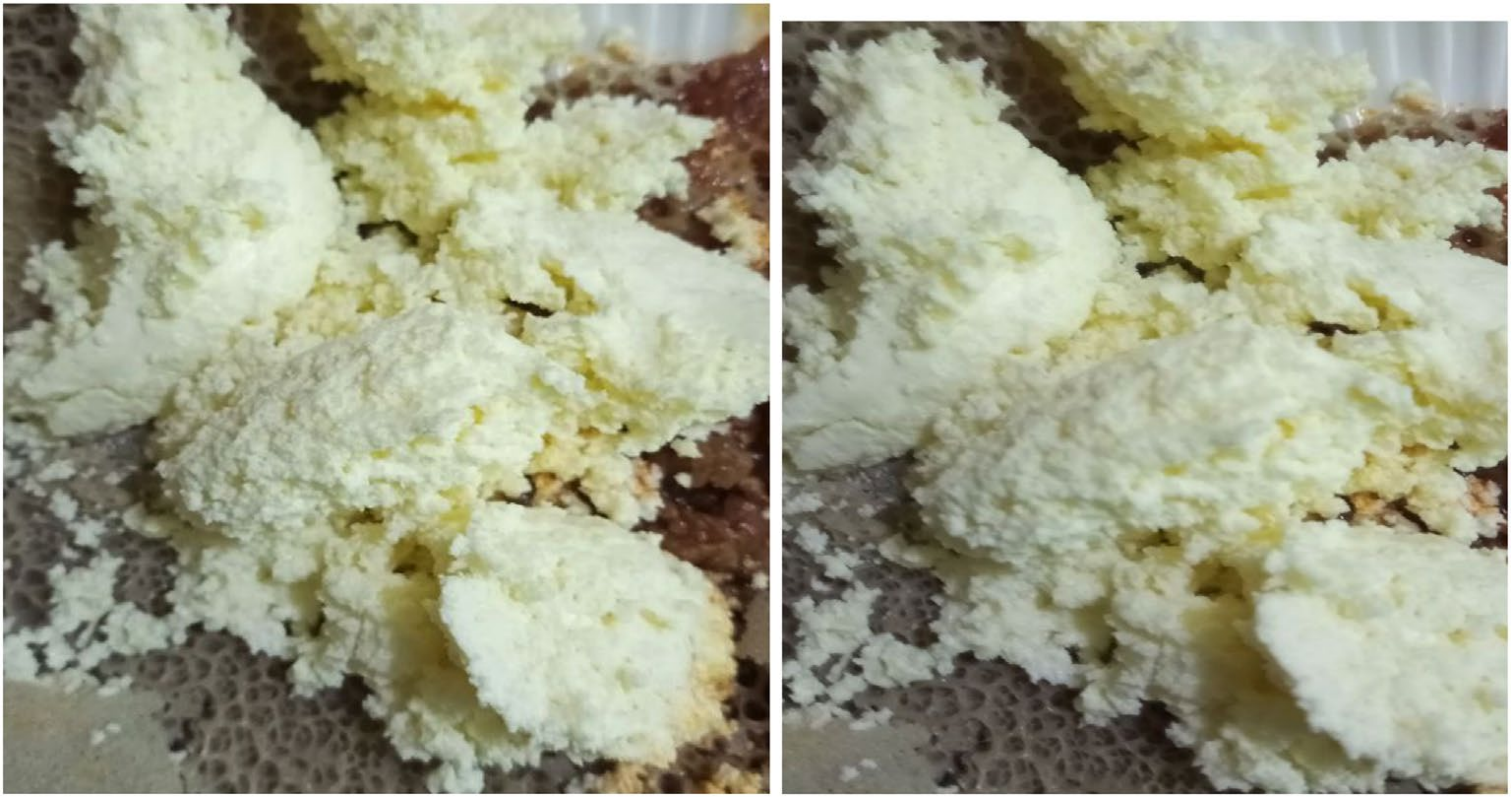
Ayib, a traditional Ethiopian cottage cheese made from fermented milk.

Ayib, a traditional cottage cheese from Ethiopia, is an integral part of the local diet and is deeply embedded in the cultural customs of many Ethiopian communities (Figure 2). Apart from providing essential nutrients such as protein, calcium, and vitamins, this fermented dairy product embodies indigenous knowledge systems and practices of dairy fermentation that date back many generations (Asefa et al. 2025). Due to the fermentation process, which extends its shelf life, milk is a useful food source in areas with limited refrigeration. Ayib's probiotic properties further emphasize its nutritional value by supporting gut health and digestion (Pillai and Morya 2026). Prepared and consumed in social settings and according to cultural traditions that support traditional values and foster interpersonal interactions (Seyoum 2025). Ayib thus underscores the importance of fermented dairy products in Ethiopian food systems and demonstrates how nutrition, culture, and indigenous knowledge can coexist (Gebremichael et al. 2025).

Ayib is formed from churned sour milk, which is used to make butter. The sour milk is churned by carefully stirring the contents of the saucepan until the butter separates. Heating the defatted milk to around 50°C causes it to begin forming a discernible curd. The curd is continuously allowed to cool before being filtered through a cotton towel (Ashenafi and Mehari 1995; Asefa and Teshome 2020), ayib is composed of 79% water, 14.7% protein, 1.8% fat, 0.9% ash, and 3.1% soluble milk constituents.

Ayib is suitable for both small children and the elderly. It is typically consumed with a variety of traditional foods, such as Kocho and Kitfo, which are traditionally made from finely ground beef (Ashenafi 2006; Sefah et al. 2024). Due to its low manufacturing costs, Ayib is one of the primary goods that are priced affordably for both domestic and local consumption (Ameye et al. 2021). Ayib is also used to make Metata Ayib, a well‐liked beverage in northwest Ethiopia. Metata Ayib is made by combining a range of locally available spices and undergoes approximately 20 days of spontaneous fermentation. It offers superior microbiological quality and a year‐long shelf life when compared to ordinary cottage cheese (Asresie et al. 2025). For instance, the rhizomes of ginger ( *Zingiber officinale* ), the garlic bulbs ( *Allium sativum* ), and the aromatic seeds of rue ( *Ruta graveolens* ) and basil ( *Ocimum basilicum* ) all have unique flavor traits that are essential to the dish (Senapati et al. 2025). The geographical knowledge and cultural heritage of these spices in Ethiopian cooking are highlighted by their Amharic colloquial names, such as “Tenadam” for Rue and “Zekakibe” for Basil. A number of these spices enhance *Metata Ayib's* nutritional profile while adding flavor. They are well‐known for their health benefits, such as garlic's cardiovascular benefits and ginger's anti‐inflammatory properties. By drawing on ancient Ethiopian culinary traditions, in which spices are masterfully combined to create complex flavors, this dish showcases the cook's talent and cultural background (Amsalu et al. 2025).

According to Ashenafi's analysis of the microbiological quality of Ayib, samples from an open market in Awassa showed notable microbial counts, including mesophilic aerobic bacteria, yeasts, and enterococci at 10^8^, 10^7^, and 10^7^ cfu/g, respectively (Ashenafi 2002). About 55% of the samples were positive for coliforms and fecal coliforms, and more than 60% had psychrotrophic levels greater than 10 log cfu/g. About 40% of the samples had a pH below 3.7, which is advantageous for limiting microbial development. The samples' pH ranged from 3.3 to 4.6. While contamination from handling and packing materials is a problem, boiling and low pH during Ayib production minimize the large initial microbial load in milk. Subsequent investigation revealed that the numbers of bacteria and yeast did not correspond to pH, with samples with pH above 4.0 exhibiting higher bacterial counts. Around 106 cfu/g of lactic acid bacteria (LAB), primarily *L. fermentum* and *L. plantarum* , were detected; however, excessive LAB and yeast growth can lead to unwanted sourness. Although low pH inhibits some pathogens, the presence of fecal coliforms indicates a potential safety risk that requires strict hygiene controls. The significance of microbial management in ensuring food safety was highlighted by (Tesfaye et al. 2011), who demonstrated that mixed lactic cultures efficiently suppressed foodborne pathogens such as Salmonella and *E. coli* during Ayib preparation and storage.

### 3.3 Arrera (Defatted Buttermilk)

Arrera is a byproduct of Ergo production, obtained during churning after butter removal. It has a viscous consistency, distinct from that of fresh milk, and is primarily composed of the casein component of milk. It is colored similarly to Ergo (Bereda et al. 2014). Arrera tastes and smells good, unlike other conventional dairy products. While it is a liquid byproduct, it is used as a raw material to make cottage cheese (Ayib) (Mohammed 2019). Commonly, mashed green *Capsicum annuum* and *Allium sativum* are added for flavor, followed by fresh leaves of *Ruta chalepensis* and *O. hadiense*. Even when smoke is added to the storage apparatus, its shelf life is only 24–48 h, which is shorter than that of any other fermented milk products (Tudu et al. 2022). Protein, leftover fat, lactose, lactic acid, milk salts, and vitamins are all present (Chengolova et al. 2024). Arrera is a popular beverage nationwide, available plain or spicy. It is provided to older adults and children of weaning age in Ethiopian rural areas, and it is especially regarded as sustenance for women and children (Temesgen 2013). Arrera is not marketed because of its relatively short shelf life and certain customary taboos or beliefs. Calves, nursing cows, and canines are thus given it when production surpasses need (Udeh 2021).

In Arrera samples from Addis Ababa, the average counts of coliforms, Enterobacteriaceae, and total bacteria were significantly high at over 9, 4.7, and 4.2 log cfu/mL, respectively (Yilma 2012). In a related study conducted in the Wolayta region, the author reported coliform counts of 4.86 log cfu/ml and total bacterial counts of almost 9 log cfu/ml (Rahel 2008). A range of Enterobacteriaceae species, including pathogens such as *Salmonella* spp., *Klebsiella* spp., and *Escherichia coli* , were reported from Arrera samples collected during the dry and wet seasons (Yilma 2012). This emphasizes the need for further research into the microbiological quality of this traditional fermented dairy product and the possible health risks associated with its consumption.

### 3.4 Aguat

Aguat is the liquid byproduct remaining after Ayib is removed from Arrera. It is a nutrient‐dense byproduct with versatile applications. Augat is an excellent choice for animal feed and a valuable source of protein for human consumption, containing about 0.75% (Berhe et al. 2017). Aguat nutritional profile is improved by its high free amino acid, enhancing its suitability for dietary and agronomic uses. Aguat can be used to reduce food waste in the Arrera production process while promoting sustainability (Zhang et al. 2023).

### 3.5 Dhanaan Fermentation

Dhanaan, a naturally fermented sour milk product, is produced by pastoralists in the eastern Ethiopian regions of Jigjiga and Shinile (Karssa et al. 2025). It possesses a five‐month shelf life and high nutritional value. Production involves placing fresh camel milk in a smoking container, covering it with a towel, and allowing natural fermentation at room temperature for 12–24 h. Similar camel milk products, such as shubat and susac, are manufactured in Sudan, Somalia, and Kenya through natural fermentation. In Sudan, raw camel milk is placed in a skin bag fastened to a camel's saddle and allowed to ferment while the camel travels, creating gariss, a fermented sour milk (Beyan et al. 2011).

Despite its economic importance, the microbiological properties and production processes of Dhanaan have not been thoroughly characterized in Ethiopia. Pastoralists prize Dhanaan for its excellent nutritional content and long shelf life (Mulaw et al. 2025). However, significant research gaps remain regarding the specific microbial consortia responsible for fermentation. Without the need for additional starter cultures, Dhanaan is made by natural fermentation. Nonetheless, some manufacturers have observed that Dhanaan can be produced in as little as 6 h by adding a small amount of previously fermented milk as a starter (Seifu 2007). Compared with spontaneous fermentation, research from Kenya shows that using specific mesophilic lactic starter cultures can improve the quality of susac, leading to a more consistent taste and a longer shelf life (Khayeka‐Wandabwa et al. 2024). By identifying which bacteria induce Dhanaan fermentation, it might be possible to standardize production processes and create a starter culture for sale. Additionally, producers emphasize the importance of keeping the milk container covered during fermentation, suggesting that the microorganisms are likely thermophilic anaerobes. possibly including thermophilic anaerobic lactic acid bacteria (LAB) and yeasts (Mulaw et al. 2025). Broad health claims are avoided in favor of describing the nutritional composition and fermentation stability. Table 1 shows fermented Milk‐Derived products, their sources, preparation, and use.

**TABLE 1:** Other fermented milk‐derived products, their source, preparation and use.

Fermented milk‐derived product	Source	Traditional preparation	Nutritional/health advantages	References
Yoghurt	Cow, buffalo, goat milk	Milk is heated, cooled, and inoculated with *Lactobacillus bulgaricus* and *Streptococcus thermophilus* ; incubated until fermentation.	Rich in probiotics, improves digestion, boosts the immune system, and is a source of calcium and protein	Abesinghe et al. (2020)
Kefir	Cow, goat, sheep milk	Milk is fermented using kefir grains containing a symbiotic culture of bacteria and yeasts; fermented at room temperature for 24 h.	Contains probiotics, improves gut health, has antimicrobial properties, and may reduce cholesterol	Azizkhani et al. (2021)
Lassi	Cow or buffalo milk	Milk is fermented with Lactobacillus species and sometimes flavored with spices or fruits; it is consumed fresh.	Probiotic benefits, aids digestion, hydrates, and is a source of vitamins and minerals.	Malik and Sheenam (2015)
Dahi	Cow or buffalo milk	Milk is boiled, cooled, and inoculated with starter culture or previous batch; incubated at ambient temperature.	Probiotic properties, improves lactose digestion, source of calcium and protein	Abesinghe et al. (2020)
Kumis	Mare's milk	Lactic acid bacteria and yeasts ferment mare's milk; traditionally fermented in animal skins or wooden containers.	Contains probiotics, rich in vitamins B and C, may improve digestion and immunity.	Mituniewicz‐Małek et al. (2025)
Cheese (e.g., Paneer, Cheddar)	Cow, buffalo milk	Milk is coagulated using rennet or acid, fermented by specific bacteria, and aged for varying periods depending on the type.	High in protein and calcium, contains beneficial bacteria, supports bone health.	Faccia et al. (2020)
Buttermilk	Cow or buffalo milk	Byproduct of butter churning or fermented milk with lactic acid bacteria; consumed fresh or fermented	Probiotic benefits, aids digestion, low in fat, source of vitamins and minerals.	Vargas‐Ramella et al. (2021)

## 4 Fermented Beverages

### 4.1 Fruit‐Based Beverages

#### 4.1.1 Tej

Tej, a honey‐based Ethiopian wine, is bitterened with “Gesho” (*Rhamnus prinoides*). It is a widely consumed beverage that is made and marketed at the household level in Ethiopia's rural, semi‐urban, and metropolitan areas. Due to differences in ingredient ratios and preparation techniques, the finished product often lacks uniform quality (Nemo and Bacha 2020). Although production has not surpassed 10% of Ethiopia's potential annual production of 500,000 tons of honey, Tej production accounts for almost 80% of the nation's honey output (Gebretsadik and Negash 2016). Tej production uses almost 80% of the nation's honey production (Gebremedhin et al. 2013). Because of its unique sensory qualities that local consumers appreciate, unrefined honey has historically been chosen for Tej over refined honey (Berhanu et al. 2026).

The first step in preparing Tej is to clean and dry the traditional fermenting container. Honey and water are mixed in a 1:3 ratio, and the mixture is allowed to ferment for two to 3 days. The boiled and cooled leaves and stems of “gesho” (*R. prinoides*) are then added to the fermented honey‐water combination. In hotter weather, this mixture ferments for an extra 8–10 days; in cooler weather, it ferments for 20 days (Steinkraus 1996). The result is ready to be served in a special glass called “Birile” once fermentation is complete. Tej fermentation is long, uncontrollable, and spontaneous because the microorganisms that ferment it originate from the raw materials, tools, and utensils. As a result, the finished product exhibits erratic sensory, microbiological, and physicochemical properties (Nemo and Bacha 2020). Because of the active yeasts, high‐quality Tej is hazy, sweet, effervescent, and yellow (Yohannes et al. 2013). The type of honey used and the amount of “gesho” added affect Tej's flavor, and the variety and number of microbes also contribute to its unique flavor (Nemo and Bacha 2020). Lactic acid bacteria (LAB) and yeasts dominate the microbial community in Ethiopian Tej, as they do in Mexican pulque (Astudillo‐Melgar et al. 2023). Tej has a very short shelf life; this is primarily due to the growth of unwanted microorganisms, which subsequently lead to over‐acidification and spoilage. Over‐acidification is the observable chemical change that renders the product unpalatable (Berhanu et al. 2023).

#### 4.1.2 Booka

Booka is a popular traditional low‐alcohol beverage in southern Ethiopia's Oromia region. The preparation procedure is easy to follow and flexible. The first step is to carefully remove the cow's bladder from a clothed carcass and clean it to remove any remaining urine. The prepared cow bladder is filled with a 1:4 mixture of water and honey. A small quantity of honey is added after the mixture has fermented for 2–3 days, and it is then allowed to continue fermenting anaerobically for two more days (Yohannes et al. 2013). The filtrate is prepared for serving as Booka once fermentation is finished. Good Booka is fragrant, sweet, and yellowish (Elema et al. 2018).

### 4.2 Cereal‐Based Beverages

#### 4.2.1 Kanyanga

Kanyanga, a traditional fermented beverage, is made through a controlled fermentation process that uses specific microbial strains to ensure consistent flavor, safety, and quality. However, there is a chance of contracting harmful bacteria if proper hygiene precautions are not followed, which raises safety concerns (Hotessa and Robe 2020). Monitoring fermentation factors such as temperature and duration is crucial to reducing risks and ensuring the final product is safe to consume (Niyigaba et al. 2025). Additionally, knowledge of fermentation dynamics may assist in optimizing the nutritional value of Kanyanga by enhancing nutrient bioavailability and producing beneficial chemicals (Singh and Kumar 2025).

In terms of nutrition, Kanyanga may contain beneficial fermentative microorganisms, particularly lactic acid bacteria, which have been associated with improved gut function. However, there are risks associated with consuming it, particularly if the fermentation process is poorly managed, leading to dangerous byproducts (Tachie et al. 2024). In addition to its nutritional importance, kanyanga is a cultural staple and a source of revenue in rural areas, making it a significant socioeconomic factor. It is commonly consumed during social gatherings and rituals, fostering a closer bond with the community and its traditions (Lyumugabe and Songa 2019). Local farmers and artisans can sustain their rural livelihoods and ensure food security in these areas by leveraging the financial opportunities that Kanyanga production and sales can offer (Kazungu and Kumburu 2023).

#### 4.2.2 Tella

**FIGURE 3:**
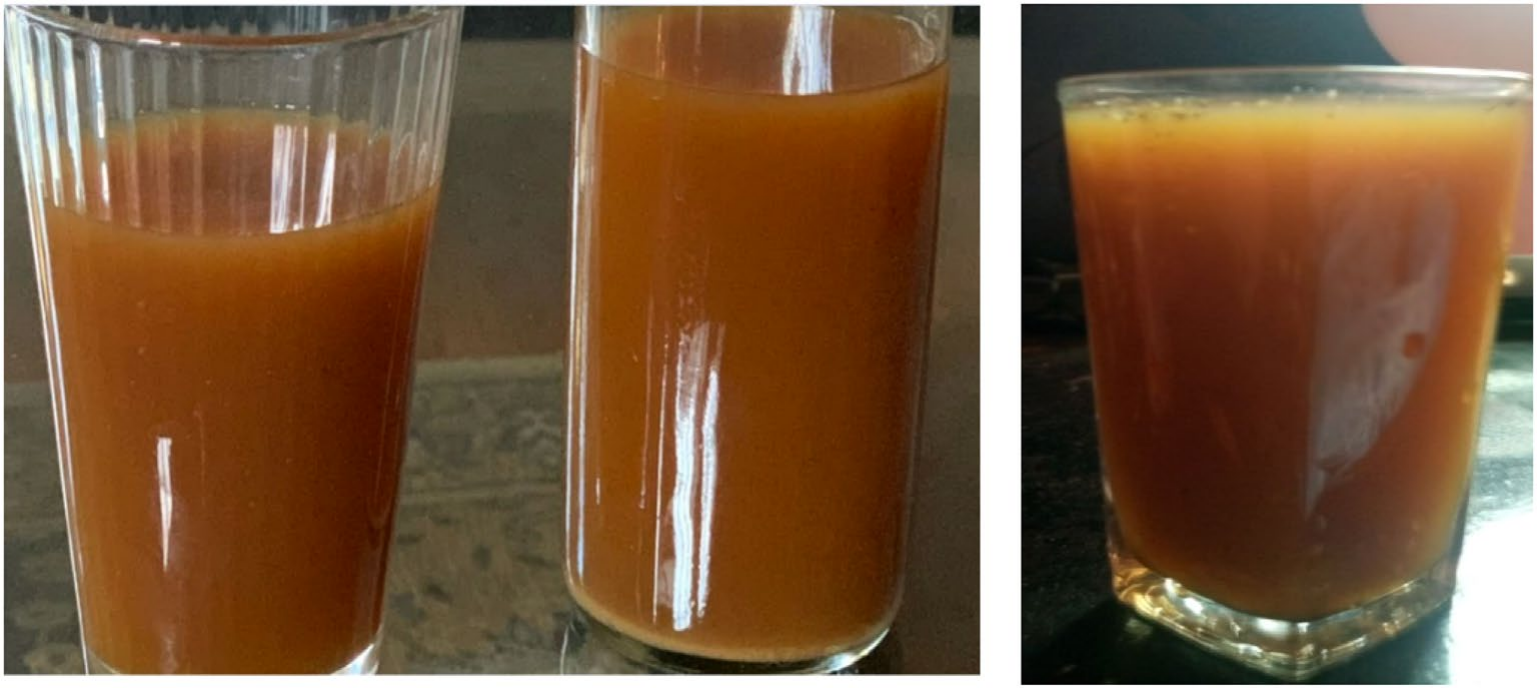
Tella, an Ethiopian known alcoholic drink with different concentrations.

Tella (Figure 3) is Ethiopia's most popular traditional fermented alcoholic beverage, particularly in the Oromia, Amhara, and Tigray regions. In addition to naturally existing microorganisms, Tella is produced from barley, wheat, maize, millet, sorghum, teff, and “gesho” leaves (*R. prinoides*) (Tekle et al. 2019). Despite high levels of production and consumption, the fermentation process remains uncontrolled, spontaneous, and unexpected (Mengesha et al. 2022). The raw materials and techniques used to create Tella vary by ethnic group, according to (Lee et al. 2015). Although there are minor variations in some places, the basic processes remain the same throughout the country.

Malt, or “Bikil,” is produced by soaking barley, germinating it, and drying it into flour. “Gesho” leaves are ground and dried. These ingredients are combined with water in a traditional bioreactor (“Insera”) to create “Tejet.” This is then mixed with unleavened bread (“ye Tella kita”) and fermented anaerobically to form “Tenses.” Finally, roasted maize flour (“Asharo”) is added to “Tenses” for a final fermentation stage to produce “Difdif,” which is filtered to yield *Tella* (Belay Berza and Awraris Wolde 2014; Tekluu et al. 2015). The supplies and equipment required to make *Tella* are the primary sources of microorganisms for the fermentation process (Tekle et al. 2019).

According to Table 2, the most prevalent fermenting microorganisms in *Tella* are yeasts (particularly *Saccharomyces* spp.), lactic acid bacteria (*Lactobacillus* spp.), and acetic acid bacteria (*Acetobacter* spp.) (Andualem et al. 2017). The pH and alcohol level of *Tella* from different places vary from 1.52% to 4.99% and 3.98% to 6.48% (v/v), respectively (Yohannes et al. 2013). *Tella* has a considerably lower alcohol level than Korean *makgeolli* (Kim et al. 2013), although it is greater than Rwanda's *ikigage*. *Tella*'s salinity, electric conductivity, and total dissolved solids (TDS) are 1.2%, 1180 mg/L, and 2359 μS/cm, respectively (Tadesse et al. 2017). *Tella* has a short shelf life of five to 7 days at room temperature because it is typically produced in homes without aseptic processing conditions. After this time, the flavor gets too sour. This sourness is mostly caused by *Acetobacter* species, which, when exposed to oxygen, transform ethanol into acetic acid (Alemu et al. 1991).

**TABLE 2:** Dominant microorganism responsible for the fermentation of traditional fermented beverages.

Beverage	Dominant microorganisms	References
Tella	Yeasts (Saccharomyces spp.), Lactic Acid Bacteria (Lactobacillus spp.), Acetic Acid Bacteria (Acetobacter spp.)	Yehuala et al. (2024)
Tej	Yeasts (Saccharomyces, Candida), Lactic Acid Bacteria (Lactobacillus spp.), Acetic Acid Bacteria (Acetobacter spp.)	Denekew et al. (2025)
Urubu	Yeasts, Lactic Acid Bacteria (Lactobacillus spp.)	Almeida et al. (2007)
Makgeolli	Yeasts (Saccharomyces spp.), Lactic Acid Bacteria (Lactobacillus spp.)	Shimoga and Kim (2021)
Ikigage	Yeasts (Saccharomyces spp.), Lactic Acid Bacteria (Lactobacillus spp.)	Lyumugabe et al. (2014)

#### 4.2.3 Borde

**FIGURE 4:**
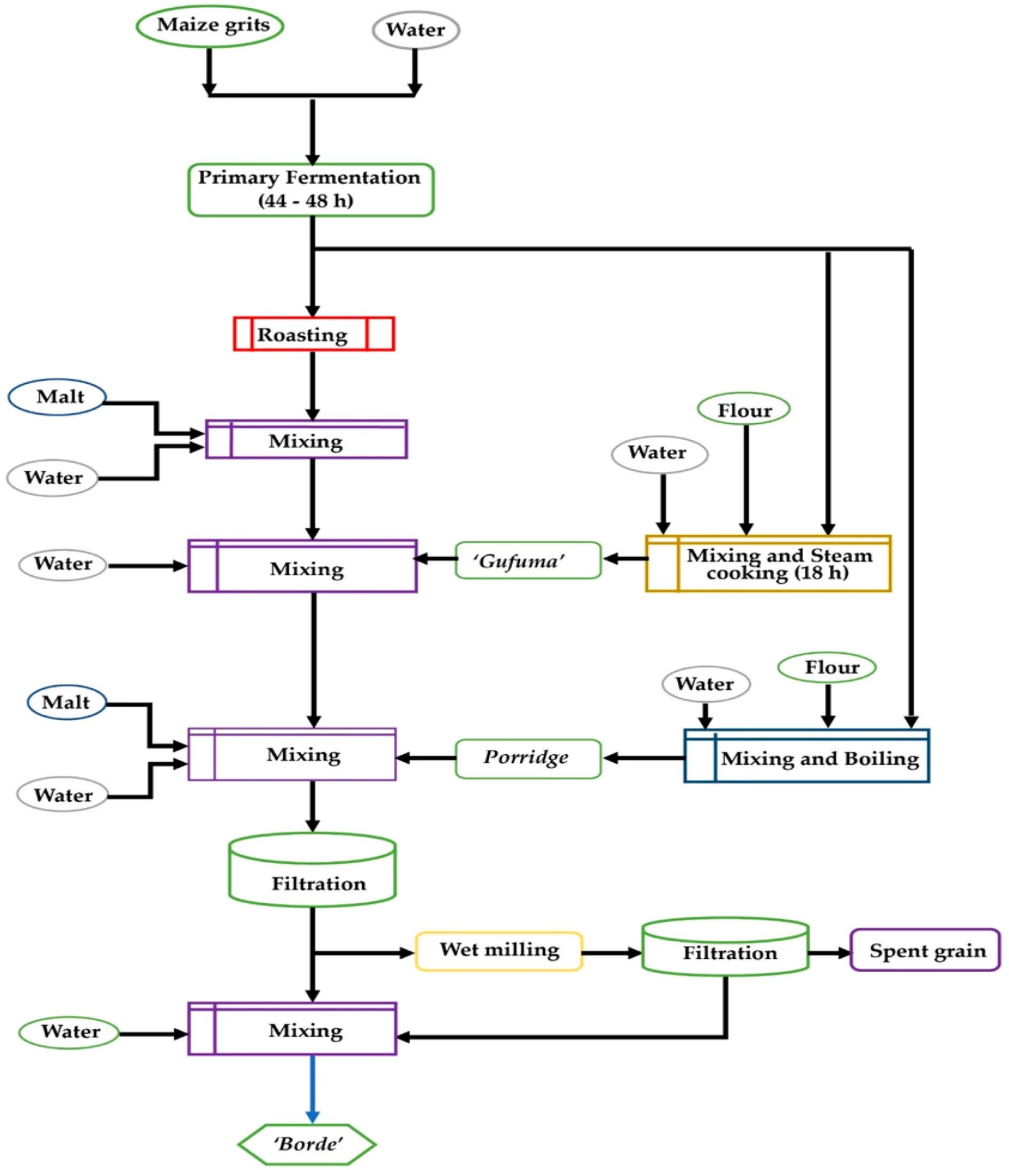
Flow diagram of Borde preparation processes (Abegaz et al. 2002a).

Cereals like finger millet ( *E. coracana* ), sorghum ( *S. bicolor* ), wheat ( *T. aestivum* ), and maize ( *Z. mays* ) can be used interchangeably or in different amounts to make borde (Figure 4), a traditional fermented low‐alcohol beverage from Ethiopia (Abegaz et al. 2002a). In Ethiopia's western and southern regions, it is widely produced and consumed. Some low‐income groups consume up to 3 L of Borde per day, and local communities often view it as a meal replacement (Abegaz et al. 2004). Borde has a high nutritional value since freshly generated batches include a large number of living cells (Hawaz et al. 2025).

Similar to *Tella*, *Borde* involves germinating barley to create malt flour. Maize grits are fermented, partially roasted into “Enkuro,” and combined with malt flour. A portion is steamed into “Gafuma” and mixed with “Tinsis” to form “Difdif.” The final mixture is filtered and diluted before serving (M. Ashenafi 2006).

Similar to the process used to prepare Tella malt, Borde is made by first germinating barley grains. Malt flour is made from this malt, which provides amylase enzymes (Ashine 2015). At the same time, a proportionate amount of water is added to the maize grits, and the mixture is fermented for around forty‐four to forty‐eight hours. Three components of the fermenting mixture are separated. A bread known as “Enkuro” is made by roasting roughly 40% of the mixture on a hot skillet, much like Uganda's kwete (Muyanja and Namugumya 2009). In the same mixing tank, the prepared “Enkuro” is combined with malt flour and additional water, and it ferments for approximately 24 h (Hotessa and Robe 2020).

To make “Gafuma,” the remaining 40% of the fermented maize grits are mixed with water and fresh maize flour, formed into a ball, and then steam‐cooked (Abegaz et al. 2002b). Then, to create “Difdif,” a thick brown mash, “Gafuma” is added to the previously made “Tinsis” (Abegaz 2007). The final 20% of the fermented maize grits are combined with more flour and water, heated to make a thick porridge, and then added to the previously made “Difdif” along with additional malt and water. Before being used as the finished product, the mixture is filtered, and a small amount of water is added to form Borde (Fentie et al. 2020).

According to (Abegaz et al. 2002a), a superior Borde is defined as opaque, effervescent, evenly turbid, gray in color, thick in consistency, smooth‐textured, and with a flavor that balances sweetness and sourness. Borde's typical pH range is 3.6 to 4.1, and variations in the finished product are due to the materials and processing conditions used (Abegaz et al. 2002b). Borde's TDS, salinity, and conductivity levels are 7139 mg/L, 1.2%, and 1180 μS/cm, respectively.

#### 4.2.4 Shamita

Many parts of Ethiopia produce and drink shamita, a traditional low‐alcohol beverage. Roasted and milled barley serves as the main fermentation substrate (Tadesse et al. 2005). For low‐income workers, this beverage replaces meals. Malt is not needed for the saccharification process in the manufacturing of Shamita, unlike other traditional Ethiopian fermented beverages such as Tella and Borde (Fentie et al. 2020).

Shamita is made by making a slurry out of barley flour, salt, linseed flour, and a few spices with water. This combination is mixed with 1 to 2 L of previously prepared slurry to create a starter culture, which is then left to ferment overnight. The beverage is ready to drink once fermentation is complete, and a small amount of bird's eye chili ( *C. annuum* ) is added (Abegaz et al. 2002a). Ashenafi and Mehari (1995) conducted the first thorough investigation of Shamita, focusing on the enumeration of microorganisms from different suppliers. Their findings indicate that lactic acid bacteria and yeasts are the most prevalent microbes in Shamita. Four years later, Bacha et al. examined microbial dynamics during Shamita fermentation and the microbial load of the raw materials (Bacha et al. 1999). Their findings showed that barley is a substantial source of fermentative bacteria, with counts approaching 10^9^ CFU/mL after 24 h of fermentation. The antibacterial qualities of the lactic acid bacteria isolated from Shamita were also examined by (Tadesse et al. 2005). They discovered that these bacteria hindered the growth of Salmonella species, including *S. flexneri* and *S. aureus* . Lactic acid bacteria from the Otiffoka region of Nigeria showed similar inhibitory effects (Temitope and Taiyese 2012). According to (Tadesse et al. 2017), Shamita's TDS, pH, conductivity, and salinity were 4520 mg/L, 3.8, 8391 μs/cm, and 4.6%, respectively.

#### 4.2.5 Korefe

In northern and northwest Ethiopia, korefe is a well‐liked fermented beverage with a foamy texture and a low alcohol content. It ferments naturally and spontaneously, just like other fermented Ethiopian drinks. Water, barley, malted barley, and “gesho” (*R. prinoides*) are the primary ingredients (Fentie et al. 2020). Korefe is made by first mixing “gesho” and water in a traditional container called a “Gan” to make a mixture called “Tijit” (Figure 2). To extract the flavor, aroma, bitterness, and fermenting microorganisms, this mixture is left for 72 h (Getnet and Berhanu 2016). Meanwhile, a dough made with water and non‐malted barley powder is baked into “Kitta,” an unleavened bread. The “Tijit,” a small amount of “Kitta,” and adequate water are combined, and the concoction is then left to ferment for around 48 h (Getachew Tafere 2015). We call the resulting semisolid combination “Tenses.”

The “Derekot,” a non‐malted roasted barley powder, is then combined with the “Tenses” and fermented for an additional 72 h. After adding water in a 1:3 ratio, korefe is ready to serve after fermenting for two to three more hours (Getnet and Berhanu 2016). According to Getnet and Berhanu (2016), Korefe has 7.01% crude fat, 2.7% ethanol, and 32 g/L titratable acidity. Korefe's salinity, conductivity, pH, and TDS are 3.7, 1.7%, 3199 μs/cm, and 1610 mg/L, respectively (Tadesse et al. 2017). After 72 h of fermentation, lactic acid bacteria and yeast counts exceed 10^9^ CFU/mL, but Enterobacteriaceae populations fall below detectable levels due to the antagonistic effects of lactic acid bacteria (Jermen et al. 2015).

#### 4.2.6 Cheka

Popular throughout southwestern Ethiopia, particularly in the districts of Dirashe and Konso, cheka is a traditional fermented beverage with a low alcohol content (Binitu Worku et al. 2018). This beverage is made with cereals and vegetables. The primary ingredients for cheka include sorghum ( *S. bicolor* ), finger millet ( *E. coracana* ), maize ( *Z. mays* ), and vegetables such as taro root ( *Colocasia esculenta* ), decne (*Leptadenia hastata*), moringa (*Moringa stenopetala*), and leaf cabbage (*Brassica* spp.) (Binitu Worku et al. 2018).

Malting is the first step in making *Cheka*, followed by the anaerobic fermentation of chopped cabbage and/or taro roots. Maize flour is added, and the mixture is fermented, crushed, and filtered. A portion is fried into “Gafuma,” combined with malt, and fermented to form “Sokatet.” Water and a thick porridge (“koldhumat”) are added for a final fermentation stage before serving (Worku et al. 2015; Binitu Worku et al. 2018). Additionally, Worku et al. (2015) reported on the nutritional value and alcohol content of Cheka, describing its physicochemical characteristics and ethanol and methanol concentrations. According to reports, the average pH, ethanol, calcium (Ca), and iron (Fe) levels of Cheka samples are 3.76, 6% (v/v), 0.2, and 0.14 mg/g, respectively.

#### 4.2.7 Keribo

**FIGURE 5:**
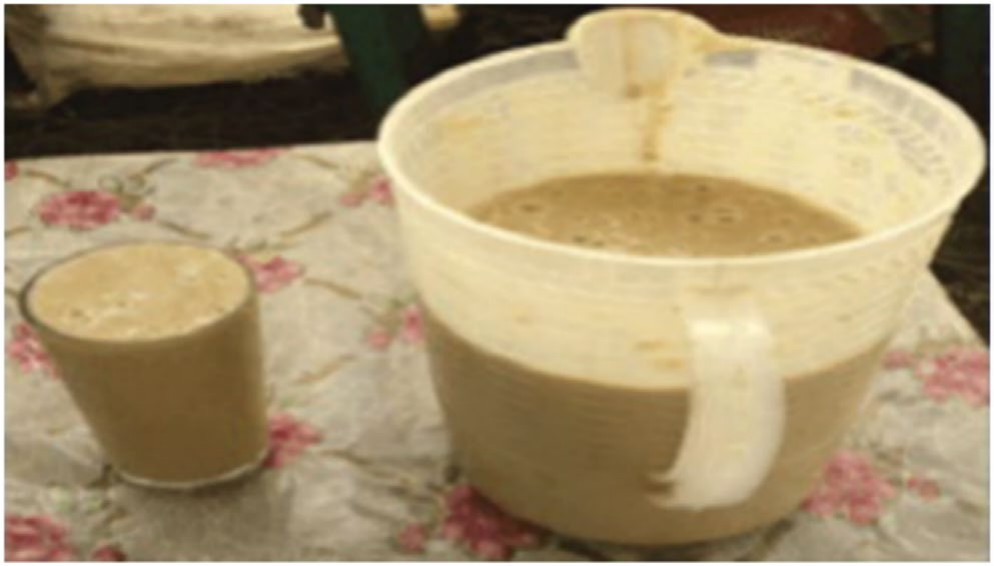
Keribo Ethiopian low alcoholic drink, especially used by Muslim people (Alemu and Kuyu 2024).

Many Ethiopians, especially those who choose low‐alcohol beverages, also love keribo (Figure 5), another traditional alcoholic beverage. The manufacturing procedure is really simple. The raw materials and processing conditions for Keribo were detailed by (Abegaz et al. 2002a). Roasted barley is first combined with boiling water, which is then cooked for roughly 20 min before the solid residue is filtered off. After the liquid has been filtered, sugar and baking yeast are added, and the mixture is allowed to ferment overnight. Lastly, before the drink is served, more sugar is added. A second paper on the microbial dynamics of Keribo fermentation was also released by (Abawari 2013). Lactic acid bacteria, aerobic mesophilic bacteria, aerobic spore formers, and yeasts had average counts of 2.70, 2.34, 4.96, and 2.01 log CFU/mL, respectively. *Staphylococci*, *molds*, and *Enterobacteriaceae*, however, had average numbers below detectable limits. Furthermore, Keribo has a maximum shelf life of 2 days at room temperature (Belay Berza and Awraris Wolde 2014).

#### 4.2.8 Areki

Like Tella, Areki is a traditional alcoholic beverage that is translucent and distilled using a more concentrated fermentation mass (Alemu et al. 1991). Yereki‐tinsis, the fermentation product, is allowed to ferment for about 5 days after a 1:2 mixture of powdered gesho leaves and powdered Bikil is made with water to create a free‐flowing slurry. Areki is separated into two categories: terra‐areki and dagim‐areki. “Dagim” means “second time,” indicating that it undergoes a second distillation, while “Terra” means “ordinary” in Amharic (Hotessa and Robe 2020). Terra‐areki is said to have an alcohol concentration of around 34.09% (v/v), with a range of 22.0%–28.0% (v/v). Terra‐areki, with a higher average alcohol concentration of almost 45% (v/v), is produced by re‐distilling dagim‐areki, resulting in a mean ethanol level of 46.6% (v/v) (Assaye et al. 2021). It is difficult to estimate Ethiopia's overall alcohol production and use because local brewed alcoholic beverages are not subject to government control (Alemu and Kuyu 2024).

## 5 Fermented Foods

Since ancient times, fermented foods have been a mainstay of human diets, serving as essential components for nutrition and food preservation (Kabak and Dobson 2011). For millions of people worldwide, the fermentation process can greatly improve food and nutrition security, particularly for vulnerable and disadvantaged groups (Mattiello and Rapetti 2024). Traditional fermentation improves flavor, palatability, and shelf life while boosting a food's nutritional content and health benefits (Satish Kumar et al. 2013). Ethiopian traditional fermented foods are often made using locally sourced ingredients and indigenous knowledge that vary by region, depending on the environment, societal norms, and consumption habits (Worku et al. 2015). In many parts of Africa, rural communities prepare and eat fermented meals derived from a range of grains, including maize, sorghum, millet, wheat, and barley (Adebo 2020). Back‐slopping allows fermentation‐related microbes to be sourced from previous batches, tools, or raw materials (Bibra et al. 2021). This process usually involves uncontrolled conditions, which could lead to variable product safety and quality. Moreover, traditional fermentation often yields small amounts of the final product and can be laborious and complex (Niyigaba et al. 2025). Tables 3 and 4 show the important fermented foods and their sources.

**TABLE 3:** Traditional fermented foods: Sources, preparation methods, and uses in Ethiopia.

Fermented food	Source	Traditional preparation	Traditional use	References
Impeke	Sorghum or maize	Grains are malted, ground, mixed with water, and fermented naturally for several days	Consumed as a traditional alcoholic beverage during social gatherings and ceremonies	Mwale (2014)
Kanyanga	Sorghum, maize, or barley	Fermentation of grains with natural yeast; distilled after fermentation to produce a strong alcoholic drink	Used as a traditional distilled spirit for celebrations and rituals	Lyumugabe and Songa (2019)
Shamita	Barley or roasted barley flour	Flour mixed with water, sometimes with spices or fruit extracts, and fermented for a short period	Consumed as a refreshing, nutritious drink, especially by laborers and farmers	Kitessa et al. (2022)
Korefe	Barley or maize	Malted grains mixed with water and fermented; sometimes flavored with local herbs or spices	Used as a traditional alcoholic beverage during festivals and social events	Alemu and Kuyu (2024)
Cheka	Sorghum, maize, or millet	Grains are malted, mixed with water and sometimes vegetables or fruits; fermented for several days	Consumed as a staple fermented beverage in southwestern Ethiopia, providing nutrition and hydration	Alemu and Kuyu (2024)
Keribo	Barley or maize	Grains are malted, mixed with water and fermented; sometimes sweetened with honey or sugar	Consumed as a mildly alcoholic or non‐alcoholic traditional beverage during social occasions	Alemu and Kuyu (2024)
Areki	Sorghum, barley, or maize	Fermented grains are distilled to produce a strong alcoholic spirit	Used as a traditional distilled liquor for celebrations, rituals, and social events	Barak (2018)
Impeke	Sorghum or maize	Grains are malted, ground, mixed with water, and fermented naturally for several days	Consumed as a traditional alcoholic beverage during social gatherings and ceremonies	Bwamu (2023)
Kanyanga	Sorghum, maize, or barley	Fermentation of grains with natural yeast; distilled after fermentation to produce a strong alcoholic drink	Used as a traditional distilled spirit for celebrations and rituals	Bell et al. (2023)
Shamita	Barley or roasted barley flour	Flour mixed with water, sometimes with spices or fruit extracts, and fermented for a short period	Consumed as a refreshing, nutritious drink, especially by laborers and farmers	Kitessa et al. (2022)
Korefe	Barley or maize	Malted grains mixed with water and fermented; sometimes flavored with local herbs or spices	Used as a traditional alcoholic beverage during festivals and social events	Alemu and Kuyu (2024)

**TABLE 4:** Major fermented foods of Ethiopia—source, preparation, and nutritional benefits.

Fermented food	Source	Traditional preparation	Nutritional/health advantages	References
Urubu	Milk	Raw milk stored in calabash gourds; naturally fermented for 2–3 days	Probiotic‐rich, improves gut flora, source of calcium and B vitamins	Oktay (2023)
Amavuta	Milk	Cream collected from fermented milk, churned into butter and clarified	High in fat‐soluble vitamins (A, D, E), energy‐rich	Buttriss (1997)
Amateregwa	Milk	Skimmed fermented milk mixed with water and stored for further fermentation	Enhances digestion, high in beneficial microbes	Agyei et al. (2020)
Urwarwa	Banana	Banana pulp mixed with water and fermented using wild yeast	Alcoholic beverage, good energy source, contains B‐complex vitamins	Wilson et al. (2012)
Isongo	Banana	Banana juice left to ferment in clay pots	Mildly alcoholic, refreshing, aids hydration	Bell et al. (2023)
Impeke	Cereals (e.g., sorghum)	Cooked cereal mash left to ferment for 1–2 days with spontaneous microbial inoculum	Increased bioavailability of minerals, natural probiotic activity	Tsafrakidou et al. (2020)
Kanyanga	Sorghum/maize/barley	Fermented mash distilled into strong alcoholic beverage	Cultural beverage, energy‐dense, but overconsumption can be harmful	Hotessa and Robe (2020)
Inyange	Cassava	Grated cassava fermented in water for 3–5 days to detoxify	Reduces cyanide content, increases digestibility	Panghal et al. (2021)
Ikivunde	Cassava	Pounded cassava wrapped in leaves and fermented underground for several days	Detoxification, flavor development, enhances mineral absorption	Masha (2022)
Imikembe	Cassava	Sun‐dried fermented cassava chips	High fiber, improved shelf life, low in antinutrients	Flibert et al. (2016)

### 5.1 Injera

**FIGURE 6:**
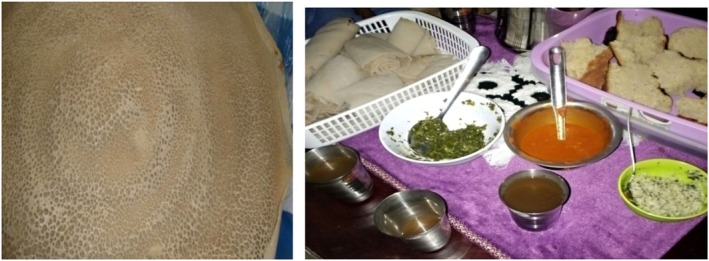
Injera, a known Ethiopian food obtained from fermented cereal crops such as teff.

The primary ingredient in injera, an Ethiopian bread resembling pancakes, is teff flour (Figure 6). This thin, fermented food is made with teff flour, water, and a beginning culture (ersho), a liquid taken from previously fermented dough (Stewart and Getachew 1962). The ideal injera has a soft texture, is thin, and has a somewhat sour flavor from fermentation (Girma et al. 2013). Throughout Ethiopia and Eritrea, injera is a staple food. Some areas also include rice, wheat, and enset ( *Ensete ventricosum* ) in their diets (Neela and Fanta 2020). Ethiopia's most popular grain is teff, which ranks third in terms of grain output and takes up around 23.42% of the country's cereal cultivation land (Taffesse et al. 2012). Given that teff is gluten‐free and hence appropriate for people with gluten sensitivity, its nutritional profile is noteworthy. Injera contributes substantially to daily intake food among many Ethiopians (Arogundade 2006).

Two phases of natural fermentation, lasting 1–3 days depending on the surrounding temperature, are required to prepare injera. Teff flour and water are first combined to create a dough, which is subsequently fermented and thinned into a batter. Poured onto a hot griddle, this batter cooks and develops its distinctive flavor and color. Because of its low pH, a good injera should taste a little sour. Injera may only be stored for 3 days at room temperature, and moldy injera is usually thrown out; it can be sun‐dried and eaten during times of food scarcity (Gashe 1985). Despite its short shelf life, injera plays a central role in household food security because it is frequently prepared and consumed.

Desiye and Abegaz (2013) collected 34 samples of injera batter at 6‐h intervals over a 96‐h fermentation period. *Pediococcus pentosaceus* and *Lactobacillus fermentum* were the most common LAB strains among the 107 LAB and 68 yeast strains found in the study. The results demonstrate that the varied microbial community involved in injera fermentation contributes to its distinct flavor and health benefits.

### 5.2 Kocho

Ethiopia's southern and southwestern regions are home to the huge, banana‐like plant known as enset ( *Ensete ventricosum* ) (Tsegaye 2002). Enset has been grown for centuries and is used for industrial fiber, animal feed, and human consumption (Tolera 2011). The Ethiopian government is actively encouraging its production in non‐enset‐growing regions to improve food security, given its drought‐resistant properties, which provide a dependable year‐round food source (Tesfaye and Lüdders 2003).

**FIGURE 7:**
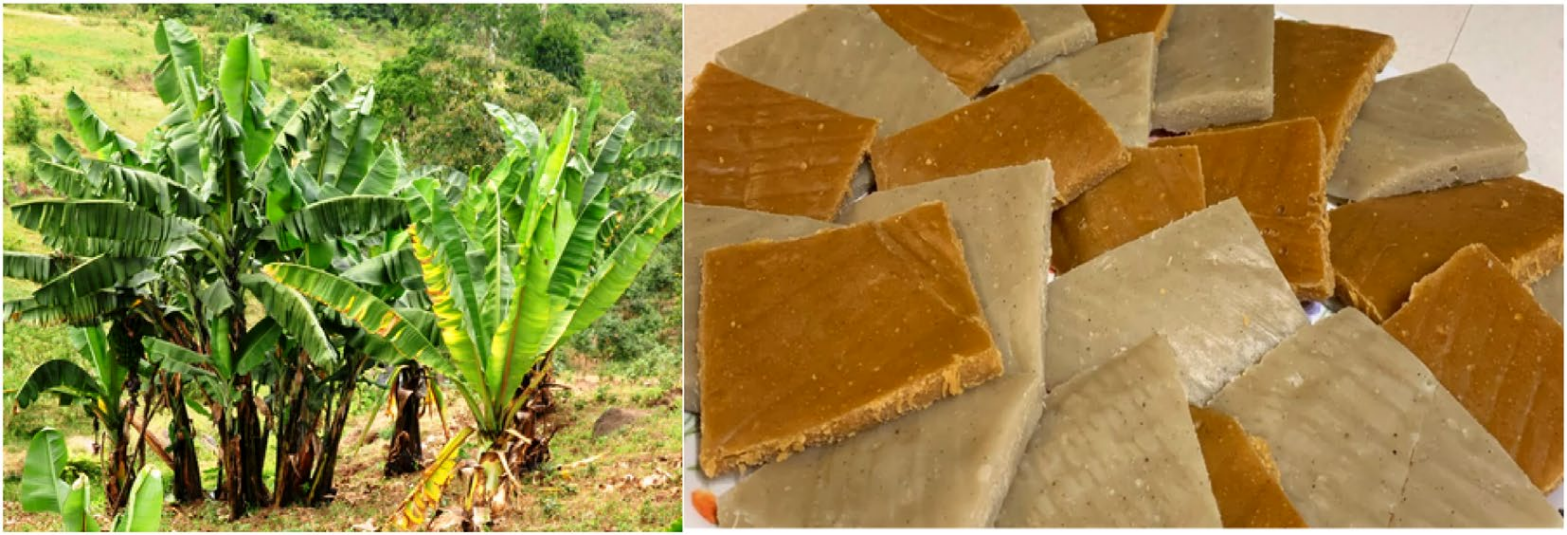
Kocho, a well‐known Ethiopian food in southern Ethiopia, is produced from *Enset* through fermentation.

The fermented food known as kocho (Figure 7) is prepared from enset by fermenting a mixture of the inflorescence stalks, pulverized corm, and scraped pulp of the pseudostem. Bulla (extracted juice) and amicho (boiled, non‐fermented corm) are two more items made from enset. In addition to aerobic and anaerobic spore‐formers, Enterobacteriaceae and yeasts, the fermentation process involves a variety of microorganisms, with LAB being the predominant group (Tiruha Habte Karssa et al. 2014).

The starter culture is made from the corms of adult enset plants. The corms are wrapped in fresh enset leaves after any unwanted pieces are removed, and then allowed to ferment for about 8 days at room temperature. There are two stages to the fermentation process: surface fermentation, lasting about 15 days, and pit fermentation, lasting another 15 days. The final product can be stored in a pit for a year. During fermentation, the activity of microorganisms that produce acid, particularly yeasts and LAB, causes the pH to decrease steadily and the number of microorganisms to increase (Andeta et al. 2018; Karssa and Darebo 2020). Studies have shown that *Lactobacillus*, *Leuconostoc, Pediococcus*, and *Lactococcus* are frequently isolated from kocho samples, indicating their significance in the fermentation process (Akalu et al. 2017).

## 6 Nutritional and Health Advantages of Ethiopian Fermented Foods

### 6.1 Probiotic Effects and Gut Health

One of the most significant benefits of Ethiopian fermented foods is their probiotic content, which may support a balanced gut flora, which is essential for digestion and immune system function. Emerging research suggests potential links to mental health (Obayomi and Edo 2025). Traditional staples like injera, made from fermented teff batter that produces short‐chain fatty acids during fermentation, contain beneficial lactic acid bacteria, such as *Lactobacillus* species (Mezemir 2015). By enhancing nutrient absorption and inhibiting the growth of harmful bacteria, these compounds may reduce the risk of gastrointestinal disorders. Frequent intake of these fermented foods has been associated with reduced gastrointestinal discomfort. Beyond the gut, these probiotics may support natural immune responses and lower inflammation, both of which improve overall health (Ji et al. 2023).

### 6.2 Nutrient Bioavailability and Antinutrient Reduction

Additionally, by eliminating antinutritional components such as phytates and tannins in grains and legumes and transforming unpalatable ingredients into more palatable forms, the fermentation process used in Ethiopian cooking may significantly enhance the bioavailability of essential nutrients (Knez et al. 2023). For instance, during the fermentation of barley for tella (a traditional beer) or teff for injera, enzymes break down complex proteins and carbohydrates, releasing vitamins like B‐complex and minerals like iron and zinc that might otherwise be poorly absorbed. In Ethiopia, where vitamin deficiencies are prevalent and plant‐based diets are the norm, this is especially crucial (Awulachew 2020).

### 6.3 Food Safety and Preservation

By removing potential contaminants, like mycotoxins in grains, fermentation acts as a natural preservative, extending shelf life without the use of synthetic chemicals and may lower the risk of foodborne illnesses. All things considered, these advantages show how Ethiopian fermented foods contribute to sustainable nutrition, offering potential recommendations for global food security and health prevention strategies (Pande et al. 2025).

## 7 Challenges and Future Prospects

Among these challenges, microbial safety and lack of standardization pose the most immediate risks to public health. The quality, flavor, and nutritional value of fermented foods produced in Ethiopia vary due to a lack of standardization, and there is no information on their potential health benefits, making it challenging to incorporate them into dietary recommendations (Woldu 2024). Additional risks include cultural barriers, such as younger generations giving up traditional customs due to modernity, and microbial safety issues with dangerous bacteria if proper fermentation processes are not followed (Inatsu and Bari 2014). Economic constraints limit small‐scale manufacturers' access to markets and resources, while regulatory issues create barriers that jeopardize product quality and safety.

Future strategies to address these issues include increasing funding for research and development to investigate the nutritional value and safety of Ethiopian fermented foods. Implementing standardized production and quality control procedures can increase consumer confidence, while awareness‐raising campaigns can highlight their health benefits. Traditional methods can be made more sustainable by assisting small‐scale producers, and their use can be promoted by including traditional fermented foods in national dietary guidelines. Initiatives to preserve cultural heritage by documenting traditional recipes and encouraging innovation in fermenting techniques will further boost the region's economic growth and nutritional security.

## 8 Conclusion

The fermented foods and beverages of Ethiopia illustrate the strong relationship between locally available substrates, traditional preparation techniques, and nutritional value. Produced from diverse raw materials such as cereals, legumes, dairy products, and honey, these traditional fermented products reflect the country's rich agricultural biodiversity and cultural heritage. The indigenous fermentation processes not only enhance flavor and shelf life but also improve nutritional quality by increasing the bioavailability of nutrients and contributing beneficial microorganisms. Despite their significant dietary and socio‐cultural importance, challenges remain in terms of process standardization, scientific documentation, and large‐scale commercialization. Strengthening research on microbial diversity, nutritional profiling, and improved hygienic practices can help preserve the authenticity of these traditional foods while ensuring safety and consistency. Integrating Ethiopian fermented foods and beverages into modern food systems and promoting them through scientific validation and public awareness can contribute to improved nutrition, sustainable food practices, and the global recognition of Ethiopia's traditional fermentation knowledge.

## Author Contributions

**D. P. Pandey:** conceptualization. **Limenew Abate Worku:** methodology, writing – review and editing. **Rakesh Kumar Bachheti:** conceptualization, methodology. **Archana Bachheti:** conceptualization. **Gyanesh Joshi:** conceptualization. **Vikas Rana:** conceptualization.

## Funding

The authors have nothing to report.

## Ethics Statement

The authors have nothing to report.

## Consent

The authors have nothing to report.

## Conflicts of Interest

The authors declare no conflicts of interest.

## Data Availability

The authors have nothing to report.
